# New perspective on maintenance therapies for platinum- sensitive recurrent ovarian cancer in women with germline and somatic mutations in BRCA1 and BRCA2 genes

**Published:** 2016-09

**Authors:** I Vergote, V Bours, B Blaumeiser, J-F Baurain

**Affiliations:** Division of Gynecological Oncology, Leuven Cancer Institute, and Department of Gynaecology and Obstetrics, KU Leuven, 3000 Leuven, Belgium.; Human Genetics Unit, GIGA-Cancer Research, University of Liège, and Genetics Center, CHU Liège, 4000 Liège, Belgium.; Department of Medical Genetics, Antwerp University and University Hospital UZA, 2650 Edegem, Belgium; The King Albert II Cancer Institute, Cliniques universitaires Saint-Luc, Université catholique de Louvain, 1200 Bruxelles, Belgiumi

**Keywords:** BRCA 1, BRCA 2, cancer, maintenance therapy, platinum-sensitive recurrent ovarian cancer

## Abstract

Ovarian cancer (OC) is the seventh most common cancer in women. Although women diagnosed with OC are usually treated frontline with platinum-based chemotherapy, most of them relapse once treatment is halted. Therefore, maintenance therapies have been developed to secure the response and delay further chemotherapy. There are two established maintenance therapies for women affected by platinum-sensitive recurrent OC: bevacizumab, a humanized monoclonal antibody targeting vascular endothelial growth factor, and olaparib, an inhibitor of poly (adenosine diphosphate [ADP]-ribose) polymerase (PARPi). Loss-of-function mutations in genes in the homologous recombination pathway, especially BRCA1 and BRCA2, predict higher rates of platinum sensitivity, better overall survival (OS), and better response to PARPi in women with OC. Among patients with platinum-sensitive recurrent OC, a BRCA mutation is the first genetically defined predictive marker for targeted therapy, since these patients are most likely to benefit from treatment with a PARPi, such as olaparib. In patients with platinum-sensitive recurrent OC without a BRCA mutation, bevacizumab currently seems to be the best maintenance option. Women with OC are progressively more routinely screened for germline BRCA mutations, and the implication of somatic BRCA mutations is increasingly being recognized in OC. Therefore, the recommendations should be updated to reflect the importance of both types of mutations. Together, these data highlight the fact that treatment of recurrent OC can be optimized using genomic contributions to individualize therapy and to improve treatment response.

## Introduction

Ovarian cancer (OC) is the seventh most common cancer and the eighth cause of cancer-related deaths in women (3.6% of cases and 4.3% of deaths). An estimated 239,000 new cases and 152,000 deaths occur every year in women worldwide (Ferlay et al., 2015). In Belgium, 766 new cases of OC were reported in 2013, and 88% of the cases occur after 50 years of age (Belgian Cancer Registry, 2013).

Almost all women with epithelial OC are treated frontline with platinum-based chemotherapy. Although a considerable proportion of women relapse after a period when treatment is halted, patients with platinum-sensitive recurrent cancer are likely to respond to further platinum-based treatment. In addition, maintenance therapy with an effective antitumor agent is used to secure and add to the response that was achieved by active treatment (i.e. platinum-based chemotherapy) and defer initiation of subsequent chemotherapy (Ledermann et al., 2014).

There are two established maintenance therapies for women with platinum-sensitive recurrent OC. Bevacizumab (Avastin®, Genentech, San Francisco, CA, USA) is an intravenously administered monoclonal antibody targeting vascular endothelial growth factor (VEGF) that alters tumour vasculature (Yoshida et al. 2015). Olaparib (Lynparza®, AstraZeneca, London, UK) is an inhibitor of poly- (adenosine diphosphate [ADP]-ribose) polymerase (PARPi). PARPis are used as maintenance therapy because they induce synthetic lethality in tumours with homologous recombination deficiency due to, for example, loss-of-function BRCA mutations. PARP is an enzyme involved mainly in the base- excision repair of single-strand errors among other DNA repair mechanisms (Benafif and Hall, 2015).

A recent study demonstrated that both germline (inherited) and somatic (acquired) loss-of-function mutations in *BRCA1* and *BRCA2* genes predict higher rates of platinum sensitivity and better overall survival (OS) in primary ovarian carcinoma (Pennington et al., 2014). Together, these BRCA mutations are present in more than one fifth of all high-grade serous OC cases (Cancer Genome Atlas Research Network 2011; Alsop et al., 2012). In view of the arrival of PARPis in clinical practice, routine testing of newly diagnosed OC patients for germline *BRCA* mutations is recommended by several guidelines. Somatic BRCA mutations are expected to be evaluated per standard testing in the near future. In view of the current available treatment options, we aim to provide an overview of maintenance therapy options for platinum-sensitive recurrent OC, including current guidelines for the treatment of patients with germline and somatic mutations in *BRCA1* and *BRCA2* genes.

## Definitions of the OC classes and their role in clinical practice

The Federation of Gynecology and Obstetrics (FIGO) surgical staging system is used for epithelial OC and primary peritoneal adenocarcinoma evaluation, and identifies the extent of the disease at the time of diagnosis (Prat and Oncology, 2014).

There are five main types of epithelial OC: high- grade serous carcinoma, endometrioid carcinoma, clear-cell carcinoma, mucinous carcinoma, and low- grade serous carcinoma (Lee et al., 2003). About 75% of women with epithelial OC have a serous carcinoma: 70% and 5% have high- and low-grade serous carcinoma, respectively (Prat and Oncology, 2014). Although low-grade serous carcinoma is less aggressive than high-grade serous carcinoma, it does not typically respond well to chemotherapy. This 2-tier grading system of ovarian serous carcinoma, which is based on defined criteria that are easy to follow, provides a good reproducibility (Malpica et al., 2004). In the future, epithelial OC will be rather classified based on the molecular profile than on histological classifications only. Endometrioid carcinomas and clear-cell ovarian carcinomas represent each approximately 10% of OCs (Prat and Oncology, 2014). Clear-cell ovarian carcinomas do not respond well to chemotherapy and may in some patients, as endometrioid ovarian carcinoma, be related to endometriosis (Jayson et al., 2014). Mucinous carcinomas account for 3% of all OCs and are often diagnosed at an early stage (Jayson et al., 2014).

## Current guidelines for germline *BRCA* mutations

A recent study suggested that germline *BRCA* mutations are found in approximately 15% of women with invasive epithelial non-mucinous OC (Alsop et al., 2012). In Belgium, multiple guidelines recommend that all women with high-grade serous or papillary epithelial OC are tested for germline *BRCA* mutations. A recent Belgian expert panel suggested to test all patients, except women affected by borderline and mucinous OC, unless they also have breast cancer (Claes et al., 2015; Robays et al., 2015).

These recommendations are reliable and are increasingly used in clinical practice. Systematic germline BRCA testing of all newly-diagnosed patients with non-mucinous or borderline epithelial OC would allow for the choice of an appropriate treatment based on the BRCA mutational status. In addition, genetic screening of all OC patients with an unknown BRCA status, who are currently receiving treatment for OC, would allow for an optimized treatment, as it has been demonstrated that about half of the patients with OC and a BRCA mutation do not have a family history of breast or ovarian cancer (Arts-de Jong et al., 2016).

## Current guidelines for *BRCA* somatic mutations

The implication of somatic mutations, which occur *de novo* in OC cells, is increasingly being recognized in OC. Somatic *BRCA* mutations have been less studied than germline mutations, but were reported in 6%–9% of patients with OC (Cancer Genome Atlas Research Network, 2011; Pennington et al., 2014).

Data on the maintenance treatment of somatic *BRCA* mutated OC patients are limited. The results of a previous study indicate a similar efficacy and safety profile of olaparib in patients having somatic and germline *BRCA* mutations (Ledermann et al., 2014). This led to the approval by the European Medicines Agency (EMA) of olaparib in both germline and somatic *BRCA*-mutated OC. The interim results of a treatment study (ARIEL2) that evaluated another PARPi (rucaparib) also suggested similar response rates in patients with germline and somatic *BRCA* mutations (Kristeleit et al., 2015; Coleman et al., 2016). Moreover, other recombination defects, not depending on *BRCA* mutation but on other mutations or loss of heterozygosity, might also prove useful to test (genomic scarring).

## Need for testing of both somatic and germline mutations

To determine the *BRCA* mutation status in a patient with OC, DNA extracted from the tumor tissue should be tested upfront to determine whether a *BRCA* mutation is present. However, oncologists should be advised to send both tumor and blood samples for testing at the same time, because the identification of germline mutations in blood samples could have familial implications.

Recently, a consensus was reached between the Belgian genetic labs. Somatic BRCA testing is currently being validated and is already available in most genetic centers. Present guidelines primarily focus on the testing of germline mutations, but in view of these recent developments, the recommendations should be updated to reflect the importance of both types of mutations.

## Maintenance therapy options for platinum-sensitive recurrent OC

**Fig. 1:**
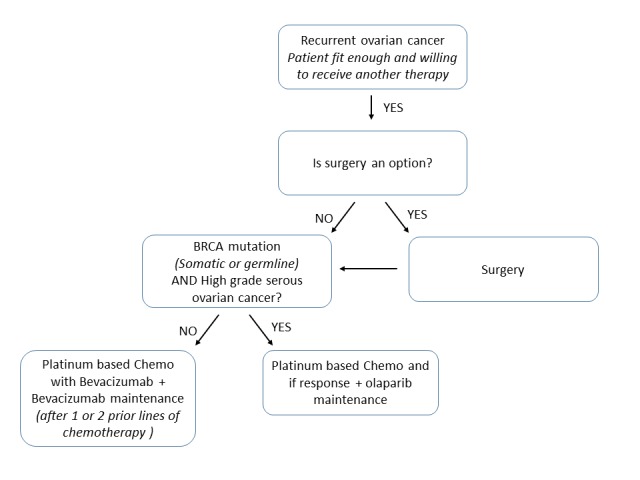
— Selection of an appropriate regimen for patients with platinum-sensitive recurrent ovarian cancer

Treatment for patients with platinum-sensitive recurrent OC can be optimized using genomic contributions to individualize therapy and to improve treatment response. Indeed, patients with high-grade serous platinum-sensitive recurrent OC, who carry a somatic or germline *BRCA* mutation, are most likely to benefit from treatment with olaparib, while bevacizumab currently remains the best maintenance treatment option in patients who do not display *BRCA* mutations (Figure 1). The main results of key clinical trials investigating olaparib or bevacizumab as maintenance treatment in platinum sensitive relapsed OC are summarized in Table I.

**Table I:** — The main results of key clinical trials investigating olaparib or bevacizumab as maintenance treatment in platinum sensitive relapsed ovarian cancer.

**OLAPARIB**						
**Study 19**		**BRCA status**	**olaparib**	**Placebo**	**HR [95% CI]**	**p-value**
Interim 1 (Lancet Oncol)^1^	**PFS**	mut	11.2	4.3	0.18 [0.10–0.31]	<0.0001
		wt	7.4	5.5	0.54 [0.34–0.85]	0.0075
	**OS**	mut	34.9	31.9	0.73 [0.45–1.17]	0.19
		wt	24.5	26.2	0.99 [0.63–1.55]	0.96
Interim 2 (ASCO 2016)	**PFS^2^**	Wt+mut	8.4	4.8	0.35; [0.25–0.49]	<0.00001
	**OS^3^**	Wt+mut	29.8	27.8	0.73 [0.55-0.96]	0.02483*
		mut	34.9	30.2	0.62 [0.41-0.94]	0.02480*
**Oza et al. 2015**			**Olaparib + CT**	**CT**		
	**PFS**	mutated	12.2	9.6	0.15 [0.34-0.77]	0.0012
**BEVACIZUMAB**						
**OCEANS ^2^**			**GC + BEV**	**GC + PL**		
	**PFS**		12.4	8.4	0.484 [0.39–0.61]	0.0001
	**OS**		33.6	32.9	0.95 [0.77–1.18]	0.65
**GOGO2013 ^4^**			**BEV + CT**	**CT**		
	**PFS**		13.8	10.4	0.61 [0.52–0.72]	<0.0001
	**OS**		42.2	37.3	0.83 [0.68–1.01]	0.056

CI, Confidence interval; HR, Hazard ratio; GC, carboplatin; BEV, bevacizumab; OS, overall survival; PFS, progression-free survival; PL, placebo; CI, confidence interval; CT, chemotherapy.

^1^ Ledermann et al., Lancet Oncol 2014; ^2^Ledermann et al., ASCO 2016 (ab 5003); ^3^ Ledermann et al.ASCO 2016 (ab 5501) ; ^4^Aghajanian et al., 2012a ; ^5^Coleman et al., Annual Meeting on women’s Cancer 2015.

*P values are deemed nominal, as study was not designed to show a statistically significant difference in OS

A retrospective analysis of data from a randomized, double-blind phase II study revealed that among patients with platinum-sensitive recurrent OC, those having a *BRCA* mutation were most likely to benefit from olaparib treatment. Overall, a longer median progression-free survival (PFS) was observed in patients treated with olaparib compared with placebo, and this difference between groups was higher in patients with a BRCA mutation compared to patients with wild-type BRCA (Ledermann et al., 2014). Although olaparib therapy was associated with a higher objective response rate and prolonged the duration of response, it did not improve the OS outcomes (Ledermann et al., 2014). Of note, this early evaluation of OS (58% of maturity) did not allow a properly powered comparison between the treatment groups, and a more mature analysis (data maturity 70%) has become recently available. Patients with a *BRCA* mutation treated with olaparib showed an OS of 34.9 months in comparison to 30.2 months when given placebo control (HR=0.62; nominal P=0.0248; study was not designed to show statistically significant difference) (Ledermann et al., 2016). In this study, the most common causes of dose interruptions or reductions in the olaparib group were vomiting, nausea and fatigue. Nausea and vomiting also tended to occur earlier, with nausea having a longer duration, in the olaparib group compared to placebo group. Serious adverse events were reported in 18% of patients in the olaparib group compared to 9% of patients in the placebo group. Adverse events causally related to treatment were recorded in 89% of patients in the olaparib group and 73% of patients in the placebo group (Ledermann et al., 2014). At the 2015 data cut-off, 15 patients remained on olaparib (of whom 8 with a *BRCA* mutation) and 1 on placebo (with a *BRCA* mutation) (Ledermann et al., 2016).

A more recent phase II, randomized, open study conducted in 173 patients with platinum- sensitive, recurrent, high-grade serous OC showed that olaparib in combination with paclitaxel and carboplatin followed by maintenance monotherapy with olaparib improved the PFS compared with paclitaxel and carboplatin alone, and that the greatest benefit was observed in patients with a *BRCA* mutation (Oza et al., 2015). A phase III trial is currently conducted in OC patients with a *BRCA* mutation to evaluate olaparib in tablet formulation as maintenance in the first-line setting (SOLO-1; NCT01844986) (Ledermann, 2016). The tablet formulation is also being evaluated in a phase III study including patients with recurrent OC (SOLO-2, NCT01874353).

In addition to olaparib, other PARPis are currently under development: veliparib (Abbvie, Chicago, IL, USA), rucaparib (Clovis Oncology Inc., Boulder, CO, USA), niraparib (Tesaro Inc., walthal, MA, USA), and the Biomarin PARPi (BioMarin Pharmaceutical Inc., San Rafael, CA, USA). Veliparib is currently evaluated in a phase III study when used in combination with carboplatin and paclitaxel, and as maintenance therapy in the first- line treatement of OC (GOG 3005; NCT02470585) (Ledermann, 2016). Rucaparib is a PARPi targeting tumors exhibiting homologous recombination deficiency and demonstrated clinical activity in heavily treated platinum-sensitive relapsed high-grade OC patients with germline mutation in BRCA. Rucaparib is now in an expanded registration- enabling study (ARIEL2; NCT01891344) for the treatment of patients with high-grade OC who have received at least three prior chemotherapy regimens (Kristeleit et al., 2015). The biomarker results from the ARIEL2 study will be applied to the analysis of ARIEL3 (NCT01968213), a phase III study in a similar population. Niraparib has also been shown to be active in patients with a *BRCA1*/*2* mutation (Sandhu et al., 2013) and is being explored in two phase III trials as maintenance therapy after first-line therapy and after response of a platinum-sensitive recurrence (NCT01847274 and NCT02655016).

Bevacizumab is the most advanced humanized monoclonal antibody agent used in platinum- sensitive recurrent OC, and remains the recommended treatment option for patients without BRCA mutations. In Belgium, bevacizumab is reimbursed as first-line treatment in FIGO stage IV epithelial OC, when administered in combination with carboplatin and paclitaxel, every 3 weeks for 6 cycles, with a maximum of 10 cycles. Thereafter, bevacizumab is given as monotherapy for a maximum of 15 months (INAMI, 2016; RIZIV, 2016). Bevacizumab in association with carboplatin and gemcitabine is also reimbursed in platinum- sensitive patients with a first recurrence of epithelial OC, and in platin-resistant ovarian cancer in combination with paclitaxel, or pegylated liposomal doxorubicin or Topotecan in second or third line, provided that they did not receive a first-line VEGF- targeted therapy (INAMI, 2016).

Bevacizumab has been shown to improve the PFS in patients with recurrent OC in three randomized phase III trials (Aghajanian et al., 2012a; Pujade- Lauraine et al., 2014; Coleman et al., 2015) (Table I). For the second-line setting, almost one-third of patients (30%) treated with bevacizumab and chemotherapy were free of disease progression after 12 months, compared with only 9% in the chemotherapy-alone arm (Aghajanian et al., 2012b). However, a survival analysis in the OCEANs trial has not brought any additional benefit in terms of OS of patients who received bevacizumab combined with gemcitabine and carboplatin compared with gemcitabine and carboplatin alone (Aghajanian et al., 2015), nor did the OS difference between the treatment arms reach statistical significance in the other two randomized trials (Pujade-Lauraine et al., 2014; Coleman et al., 2015). Hypertension and proteinuria were the most common adverse events in both the OCEANs and AURELIA ENGOT-ov4 trials, and the most common adverse events leading to study discontinuation together with neutropenia and epistaxis in the OCEANs trial (Aghajanian et al., 2012a; Pujade-Lauraine et al., 2014). In the final analysis of the OCEANs study, hemorrhages outside central nervous systems were recorded in 68.0% of patients in the bevacizumab and carboplatin group compared to 32.6% of patients in the carboplatin and placebo group (Aghajanian et al., 2015). In the AURELIA ENGOT-ov4 study, adverse events possibly related to tumor burden were less common in the bevacizumab group, whereas hand-foot syndrome and peripheral sensory neuropathy was more common in the bevacizumab group (Pujade- Lauraine et al., 2014).

In addition to the three randomized phase III trials, a recent case-control study has been conducted in patients with advanced OC receiving carboplatin and paclitaxel with or without bevacizumab as first-line treatment. In this study, time to progression after the secondary chemotherapy was 3 months shorter in women receiving carboplatin and paclitaxel with bevacizumab compared with the control group (Petrillo et al., 2016). when the disease recurred in these patients, it was more widespread in those who received bevacizumab-containing first-line chemotherapy, with multiple anatomic sites and wider diffusion of peritoneal disease. This study suggested that while including bevacizumab in upfront regimens prolongs platinum-free interval in advanced OC patients, it is associated with more aggressive behaviours of recurrent disease (Petrillo et al., 2016). Possible factors that contribute to the resistance to bevacizumab include the activation and/or upregulation of alternative pro-angiogenic signaling pathways and recruitment of bone marrow-derived pro-angiogenic cells and pericytes that modify the tumor microenvironment, thereby obviating the need for VEGF signaling (Marchetti et al., 2015). Moreover, a number of patients with recurrent OC are no good candidates for bevacizumab treatment due to a history of bowel obstruction or bowel infiltration (Burger et al., 2014).

In summary, olaparib should be considered as the treatment of choice as second-line treatment option in patients with platinum-sensitive recurrent OC who carry a somatic or germline BRCA mutation and treatment should be initiated as soon as second line management is considered appropriate. In contrast, bevacizumab, in combination with carboplatin and gemcitabine, can be considered for patients with platinum-sensitive recurrent OC who do not display BRCA mutations. A full detailed decision tree for patients with platinum-sensitive recurrent OC is presented in Figure 1.

## Conclusion

A problem for health-care systems is that most drugs used to treat recurrent OC are expensive and it is unclear which patients will benefit from treatment. PARPis have shown to be most active in patients with a *BRCA* mutation. A *BRCA* mutation is therefore the first genetically defined predictive marker for targeted therapy of OC. The availability of PARPis as treatment option for germline and somatic *BRCA* mutated patients opened the door for routine testing of mutations in blood and in the tumor (Marchetti et al., 2015). In patients without a *BRCA* mutation and a platinum-sensitive recurrence, bevacizumab currently seems to be the best maintenance option.

## Future directions

A change in the timing of PAPRi administration may be beneficial. The results of a previous study suggested that patients should be screened for *PARP1* expression prior to therapy with PARPi, and that treating chemotherapy-naïve patients with PARPi prior or concurrently to the administration of chemotherapy may increase the responsiveness to PARPi (Marques et al. 2015). A randomized, double- blind, phase III trial (PAOLA-1, NCT02477644) is currently ongoing in patients with advanced high- grade serous or endometrioid OC to evaluate the effect of olaparib combined with platinum-taxane chemotherapy as first-line treatment followed by a maintenance treatment with olaparib.

In addition to *BRCA1*/*2*, other genes, including *ATM*, *BRIP1*, *CHEK1*, *CHEK2*, *NBN*, *RAD51C*, and *RAD51D*, play key roles in homologous recombination and could also confer sensitivity to PARPi (McCabe et al., 2006; Loveday et al., 2011; Walsh et al., 2011; Hodgson et al., 2015; Norquist et al., 2016). The growing importance of PARPis as therapeutic agents adds incentive to better characterize the role of PARPis in this subset of ovarian carcinoma.

As maintenance therapies are often added to a frontline therapy, sensitivity to platinum-based chemotherapy is no longer measured, and rather the effect of the maintenance therapy itself is assessed.

## Trademark statement

FoundationOne® is a registered trademark of Foundation Medicine
